# Barriers driving health care disparities in utilization of age-appropriate screening

**DOI:** 10.3389/fpubh.2023.1100466

**Published:** 2023-03-03

**Authors:** Amany R. Keruakous, Noha Soror, Sarah Jiménez, Rachel Ashley, Mai Keruakous, Betro T. Sadek

**Affiliations:** ^1^Department of Internal Medicine, Georgia Cancer Center, Augusta University at Medical College of Georgia, Augusta, GA, United States; ^2^Section of Hematology-Oncology, University of Oklahoma Health Sciences Center, Oklahoma City, OK, United States; ^3^Mercer County Community College, West Windsor, NJ, United States; ^4^Prisma Health, Columbia, SC, United States

**Keywords:** racial disparities, cancer care, cancer screening, racial minorities, socioeconomic status

## Introduction

Cancer is one of the leading causes of death worldwide. It continues to be the second leading cause of death in the United States despite all national efforts aiming to reduce cancer burden and mortality. Per the Centers of Disease Control and Prevention (CDC), 602,350 deaths in the United States were attributed to cancer in 2019 (1). Improving cancer screening metrics is a crucial healthcare goal, with emerging data showing a significant reduction in compliance rate to cancer screening throughout the U.S., specifically amid overwhelmed healthcare system due to the current COVID-19 Pandemic (2). Cancer screening is an essential element of early cancer detection. Early diagnosis is a key determinant of timely treatment and survival outcomes for cancer patients and remains a cost-effective means of reducing cancer mortality. Delays in medical care and age-appropriate screening lead to increased cancer burden, reflecting the overall prognosis. In the United States of America, one-third of the adult population has cited challenges in medical care accessibility.

According to the current United States Preventive Task Force (USPSTF) recommendations, biennial screening mammography is recommended for women aged 45–74 years (3). For colorectal cancer screening, the USPSTF recommends screening in all adults aged 50–75 years (4, 5). The USPSTF recommends screening all females aged 21–29 every 3 years with cervical cytology alone for cervical cancer screening. For women aged 30–65 years, the USPSTF recommends screening every 3 years with cervical cytology alone, every 5 years with high-risk human papillomavirus (hrHPV) testing alone, or every 5 years with hrHPV testing in combination with cytology (co-testing) (6).

Adults aged 50–80 years who have a 20-pack-year smoking history and currently smoke or have quit within the past 15 years are recommended to be screened for lung cancer with low-dose computed tomography (LDCT). However, for prostate cancer screening for men aged 55–69, periodic prostate-specific antigen (PSA)-based screening for prostate cancer is recommended to be individualized (7).

Unfortunately, these cancer screening tools are not distributed equally across the U.S. population, with evidence of health care disparities in economically disadvantaged regions (8, 9).

Despite Medicaid expansion, which increased health insurance coverage and improved access to healthcare services for low-income Americans (10), racial and ethnic disparities are still problematic, with a wide gap in the healthcare system (11, 12).

Appropriate interventions to maximize screening in racial/ethnic minorities must be targeted toward the most vulnerable patient populations.

## Aims

This Perspective aims to report the sociodemographic healthcare disparities and the challenges in medical services accessibility due to financial burden. Identifying the barriers to utilizing age-appropriate screening is key to delivering a more precise approach to overcoming healthcare disparities.

## Methods

Using the Behavioral Risk Factor Surveillance System (BRFSS) questionnaire, we conducted a descriptive summary of 2020 BRFSS survey respondents. Data were analyzed to report the healthcare disparities of respondents to the 2020 BRFSS survey. We reviewed responses from participants who failed to have age-appropriate screening for breast and colon cancer.

## Results

Factors identified to explain the advanced stage of cancer diagnosis and higher cancer mortality in underserved communities include lower socioeconomic status, lack of access to health care, lower rates of routine screening, and a lower likelihood of receiving recommended treatment for cancer (13).

We noticed from the BRFSS responses that among oncology patients, there was a higher proportion of unemployment, lower level of education, and lower annual income. However, the majority of them had health care coverage.

We report the impact of medical care's financial burden on cancer patients and its association with racial and socioeconomic disparities. We divided the patients based on their responses to the question, “Ever had difficulty seeing a physician due to medical cost?” and stratified the respondents based on racial and socioeconomic factors.

The proportion of participants who have difficulty seeing a physician due to cost varies between race and ethnicity. Multiracial minorities are approximately three times higher than the White race population to report the financial burden. In addition, the annual income, education level, and employment status are essential factors, with a higher financial burden among the lower annual income group (< 25k) and among participants who did not finish high school. African American participants had a higher percentage of lower annual income and higher unemployment rates.

We report compliance rates to age-appropriate screening and its association with socioeconomic status. There were differences between the groups of participants who delayed medical care due to cost vs. not; we reported lower compliance rates to screening mammography and colonoscopy for patients who reported financial difficulties accessing medical care.

The compliance with screening colonoscopy was affected by accessibility to medical care and medical cost; additionally, it was associated with the participants' race. For all cancer patients, participants of Asian, Hispanic, American Indian, and multiracial descent had higher non-compliance rates when compared to White or African-American descent. The non-compliance rates were even higher among the financially burdened population, across all races, with more pronounced differences in the Asian and Hispanic participants.

The responses for screening mammography are better, with lower rates of non-compliance across all races, compared to colon cancer screening. Moreover, there is still room for improvement among Hispanic and multiracial descent. The same observation is consistent for screening mammography, with higher rates of non-compliance among participants who reported delays in medical care due to cost.

We report that compliance with age-appropriate screening tools is lower among participants who had difficulties accessing medical care due to overbearing medical costs. We could determine that securing a healthcare coverage plan for the patients is critical to remediate compliance.

## Discussion

There is a good body of evidence that improving health care coverage reflects on the screening rates and compliance (14–16).

Not only healthcare coverage but racial and socioeconomic disparities and level of education are crucial to delays in medical care. Future research and interventions should target those minorities to improve cancer care.

Medicaid expansion in certain states caused more significant improvement in total insurance and Medicaid coverage and mammogram rates in lower-income women than in non-expansion states after Medicaid legislation was passed. Studies demonstrated that people take advantage of expanded eligibility by acquiring insurance, which can improve access to preventive measures, such as screening mammography and colonoscopy (14, 15).

Adults with low income may lack insurance coverage through work but earn income just above the minimum amount needed to qualify for governmental low-income insurance programs, such as Medicaid (17, 18).

Unemployment is a significant factor evident among all racial classes in the U.S., which is more pronounced with the COVID-19 pandemic and our aging cancer population. COVID-19 pandemic has an adverse employment change and may have harmful impacts on workers' mental health, with disproportionate effects on racial minorities (19–21). The unemployed patient population is expanding, and efforts should be directed toward improving cancer care. There is a higher proportion of unemployment among the African American population.

Cultural perceptions are also barrier for age-appropriate cancer screening. The general attitude toward health care can be a challenge for appropriate cancer screening. Cultural perceptions can also affect patient decisions more than healthcare providers realize. For example, the diagnosis of “cancer” carries a stigma in many cultures (22). This can be related to associations and cultural believes that cancer is attributable to some form of bad luck or will of a supreme power. Therefore, it is crucial for healthcare providers to understand that such cultural perceptions of cancer can negatively impact screening rates and perception of importance of such preventive measures (23, 24).

Other religious values, and cultural practices that can hinder appropriate cancer screening can include, a false perception that the risk of cancer is low in certain ethnic groups, faith in traditional remedies, or viewing cancer as a sentence of death (25). Other specific cultural barriers to cancer screening for pelvic and breast malignancies is the cultural perception of modesty among women or concerns about preserving sexual function in men in certain cultural backgrounds (24). Lack of printed translated brochures/ patient education materials, lack of trust of the healthcare system and receiving less provider recommendation for cancer screening are more exacerbated by health care providers who fail to fully estimate and overcome those barriers (26, 27).

Cultural barriers might also make certain groups to be less information-seeking, and less accepting of screening for disease in the absence of symptoms compared to other groups (24).

Another factor that can affect recipients of appropriate screening is the out-of-pocket costs, even in population categories that are not considered low-income despite being insured. Dorn et al. examined colorectal cancer screening and observed that those with insurance reported significant declines in colonoscopy despite being insured in those with high out-of-pocket costs (28).

Other studies investigated racial disparities in breast cancer screening in A.A. women with findings of increased mammography rates. The authors postulated that the increasing mammography rates in this category might be due to a higher percentage of A.A. women having qualified for public insurance (29), which is another resource for racial minorities.

Other factors that potentiate racial inequalities include quality of housing, access to healthy food, transportation needs, and violence. Low awareness of screening guidelines, lack of appropriate access to information about the availability of new screening tools, cultural misbeliefs about screening, understanding of the risk, the stigmata of cancer, and the lack of access to facilities with cancer screening programs were all factors that negatively impacted colorectal screening (30). These factors altogether can widen the gap in access to health care (31).

Addressing these critical gaps in healthcare and screening tools access requires better data collection on racial inequalities in the healthcare system, increased research on racial and socioeconomic-sensitive policies, increased awareness of these disparities among healthcare professionals and leadership personnel in positions of implementing healthcare policies, and finally involving racial minorities in the development of these practice-changing policies.

## Summary and recommendations

In summary, our Perspective summarizes health disparities and the barriers to compliance with screening tools. Due to the financial burden, delayed medical care is more pronounced in racial minorities, low socioeconomic status, and unemployed patients and should be targeted in future quality improvement projects. Given the harmful effects of delayed diagnosis and treatment of cancer, public health and clinical professionals must utilize tools to improve cancer screening accessibility to minorities with socioeconomic and racial disparities.

## Author contributions

All authors listed have made a substantial, direct, and intellectual contribution to the work and approved it for publication.
